# A Novel Approach to Support Failure Mode, Effects, and Criticality Analysis Based on Complex Networks

**DOI:** 10.3390/e21121230

**Published:** 2019-12-16

**Authors:** Lixiang Wang, Wei Dai, Guixiu Luo, Yu Zhao

**Affiliations:** 1 School of Reliability and Systems Engineering, Beihang University, Beijing 100191, China; wlx29@buaa.edu.cn (L.W.); zhaoyu@buaa.edu.cn (Y.Z.); 2 Nanjing Chenguang Group Co., Limited, Nanjing 210006, China; pengchang_jiang@126.com

**Keywords:** complex network, failure mode and effects and criticality analysis (FMECA), entropy centrality, influential nodes

## Abstract

Failure Mode, Effects and Criticality Analysis (FMECA) is a method which involves quantitative failure analysis. It systematically examines potential failure modes in a system, as well as the components of the system, to determine the impact of a failure. In addition, it is one of the most powerful techniques used for risk assessment and maintenance management. However, various drawbacks are inherent to the classical FMECA method, especially in ranking failure modes. This paper proposes a novel approach that uses complex networks theory to support FMECA. Firstly, the failure modes and their causes and effects are defined as nodes, and according to the logical relationship between failure modes, and their causes and effects, a weighted graph is established. Secondly, we use complex network theory to analyze the weighted graph, and the entropy centrality approach is applied to identify influential nodes. Finally, a real-world case is presented to illustrate and verify the proposed method.

## 1. Introduction

The formation of product quality runs through the whole process of product development, from product design and machining to assembly. Improving performance of products and reducing the failure of them has always been the common pursuit of academia and industry. However, due to the random fluctuation of the process factors, deviations in quality characteristics occur, and variations in quality characteristics lead to defects as time goes on, which may cause product failure. As we all know, the failure of products may be a destructive blow to the company’s production and operation. Therefore, it is crucial to identify the failure modes that may exist on products. Hence, the possible effect of any failure mode shall be analyzed, so as to prevent it from happening and reduce its losses. However, with an increasing complexity of current products, a number of products are always composed of several subsystems [1,2] and interaction between each subsystem in order to carry out system function [3]. Moreover, for complex products, the components in the subsystem have many quality characteristics, which are coupled to each other, and different coupling modes make the failure modes in products diverse. In addition, products are affected by various external factors in the design, development and production process. In the Internet of Things (IoT), many devices are connected together by outside resources [4]. If a device fails, the Internet of Things devices may not function properly at the network layer. These make the identification and importance ranking of failure modes in products more and more difficult.

One of the main identification and importance ranking methods of failure modes is the failure mode, effects and criticality analysis (FMECA). FMECA is a reliability analysis method which discovers the potential failure modes in a system, so as to evaluate the effects on system performance [5]. The method consists of two parts: failure mode effect analysis (FMEA) and criticality analysis (CA). On the basis of the CEI EN 60812 standard [6], FMEA is a systematic procedure for the analysis of a system to identify potential failure modes, and their causes and effects on system performance. On the other hand, Criticality Analysis plans and focuses the maintenance activities according to a set of priorities by giving failures with the highest risk the highest priority [7]. Carrying out a typical FMECA, a complex product can be considered as a system, and the system is divided into several subsystems based on the fundamental principle of functional independence. Then, the subsystem is divided into component level, layer by layer, until the level of single component. FMECA starts from the indenture lowest level (single component) and continues analyzing the upper hierarchical level. According to the importance of the failure modes, FMECA assigns different priorities for taking countermeasures. The higher the importance of the failure mode is, the higher its priority. In the traditional FMECA, importance of each failure mode is ranked based on the risk priority number (RPN), which is derived by the product of three risk factors: occurrence (*O*), severity (*S*) and detection (*D*) [8]. Therefore, FMECA is a good way to simplify and solve complex product reliability and continuously improve product performance. It has also been widely applied in a range of fields, such as machine tool [9], healthcare field [8], wind industry [10] and nuclear industry [11]. However, the classical RPN formula has highlighted many drawbacks in analyzing practical problems [12,13]. Further, FMECA ignores the association and influence between failure modes, and their causes and effects, and fails to fully exploit failure information. Obviously, as mentioned above, due to its functional coupling, complex structure and numerous parts, classical FCECA is difficult to solve the importance ranking of failure modes in complex products. Therefore, the failure coupling information between products, components and parts must be fully mined to analyze the failure mode more accurately.

Thus, this paper introduces the complex network theory that defines failure modes, and their causes and effects as nodes. The logical relationship between failure cause and failure mode are defined as the edge, and the weight of the edge is represented by the square root of occurrence (*O*) multiplied by detection (*D*). Analogously, the relation between failure mode and failure effect is denoted as edge, and the weight of the edge is represented by severity (*S*). Then a weighted graph is established. Furthermore, the entropy centrality approach is applied to identify influential nodes. Finally, a real-world case is presented to illustrate and verify the proposed method.

The contributions of this paper can be summarized as follows.

(1) Complex network theory is introduced into FMECA, and a weighted graph is established to analyze the influence relation between failure modes, and their causes and effects.

(2) Entropy centrality is used to identify the vital nodes of a weighted graph, and the weight of the network is mutative; moreover, the number of nodes in the network is also changing.

The remaining sections of the paper are organized as follows. Section 2 reviews the literature on FMECA improvement and identifying influential nodes. Section 3 includes details of the proposed approach and gives a summary of the method. Section 4 contains details about a real-world case to illustrate and verify the proposed method. We conclude our work in Section 5.

## 2. Literature Review

This article is mainly related to two streams of literature. The first one is the literature on FMECA improvement. The traditional FMECA uses Risk Priority Number (RPN), which given by the multiplication of the risk parameters *O*, *S*, *D* of a failure to quantify the risk of failure modes [6,14].

(1) RPN = O⋅S⋅D

In Equation (1), *O* defines the probability of a failure mode will come out, *S* indicates the degree of failure mode effect system, *D* implies the probability of failure mode has been identified before the system is affected, and each risk factor generally takes a discrete value in the range [1,10].

Despite its wide use, the classical RPN has many shortcomings, which have been highlighted countless times, such as the presence of gaps in the range of admissible values and the high sensitivity to small changes [7,15,16,17].

Several authors proposed some methods to deal with the restriction of RPN. Braband adopts the sum of the three parameters to improve RPN assessment, which is called IPRN [18]. An exponential RPN (ERPN) was proposed by Chang et al. to reduce the number of duplicates RPNs [19]. To make the ERPN better, Akbarzade Khorshidi et al. came up with URPN [20]. Both ERPN and URPN well-solved the trouble of duplicates RPNs and of relative importance among *O*, *S* and *D*. Carmignani suggested the use of a fourth parameter in the RPN calculation [21]. The profitability based on costs and possible profits after minimizing losses due to failure is taken into consideration. Except the above optimization of RPN, most of the work is mainly focused on fuzzy logic, for the classical FMECA ignores some real situations. Hence, fuzzy FMECA methods are employed to express the uncertainty [22,23]. The approach needs a large amount of proper expertise and experience. Thus, the use of evidence theory [24] is presented to manage the uncertainty and support to characterize this type of evaluation.

Rathore et al. [25] discussed how a selection with hesitant fuzzy information is a multi-criteria decision-making problem. The multi-criteria decision method (MCDM) is also frequently used to support FMECA. Braglia [26] proposed the Analytic Hierarchy Process (AHP), which uses the classical risk parameters *O*, *S* and *D* along with the expected cost due to failure as a standard to compare the potential causes of failures. Braglia et al. [27] also adopted the fuzzy technique for order preference by similarity to ideal solution (FTOPSIS) method to prioritize the potential risks of failure modes in criticality analysis. A combined FTOPSIS and fuzzy-AHP [28] approach to FMECA is proposed by Kutlu and Ekmekçioğlu [29]. Based on FTOPSIS and AHP, a decision support tool is proposed by Carpitella et al. to perform a reliability analysis with relation to a subsystem, in which the consensus obtained by modeling the different decision-making capabilities of each expert is not taken into account in the process of judging from the experts. Liu et al. provided an algorithm to cope with the group decision making characterized by the large number of participators in distributed groups and based on conflict assessments and majority opinions [8].

The second related stream of research is on complex network theory. Complex network theory is an effective method to analyze system complexity, and it has been widely applied to many fields, such as social networks, and biological networks [30,31]. It is crucial to identify the most influential nodes in complex networks for optimizing the network structure and accelerating information dissemination. In network analysis, measuring centrality is one of the important ways to identify the most influential spreaders. Degree centrality (DC) [32] is a basics measure, and the importance of one node is measured by the number of its neighbors. Global measures such as betweenness centrality (BC) [33] and closeness centrality (CC) [34] can identify node influences in the global scope. Kitsak [35] proposed a new centrality measure called “k-shell decomposition”. This measure, which determines the centrality of nodes based on their locations in the network, considers nodes topographically located in the core of the network as influential nodes. Katz [36] introduced a measure of centrality known as Katz centrality, which computed influence by taking into account the number of walks between a pair of nodes. Stephenson and Zelen [37] defined the information centrality using the “information” contained in all possible paths between pairs of points. Ahmad Zareie et al. [38] have taken advantage of an entropy-based approach to detect the spreading capability of nodes in networks on the basis of their topological information.

## 3. Proposed Method

### 3.1. Preliminaries

The FMECA worksheet contains a wealth of product failure information, but it can only meet the needs of simple information retrieval. The information mining of failure modes, and their causes and effects are the descriptions of quality features of products from multi-level, which can be transformed between different levels.

**Figure 1:**
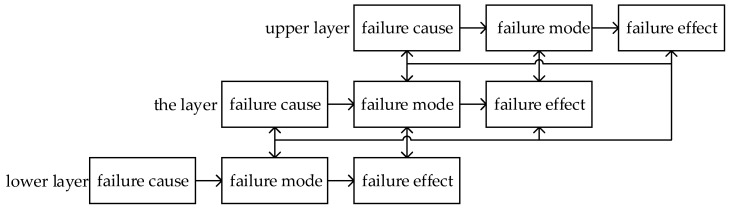
Transformation and logical relation between failure modes, and their causes and effects.

FMECA denotes that the cause of failure is failure mode in the lower layer, and the effect of failure is failure mode in the upper layer. The transformation and logical relation between failure modes, and their causes and effects, are shown in Figure 1.

Where failure cause leads to failure mode break out, failure mode has three different effects on the product function, which are the local influence on the layer, the high-level impact on the upper adjacent layer, and the final impact on the initial indenture level. We introduce a complex network to describe the coupling relations between failure modes, and their causes and effects. Based on Figure 1, we consider failure mode, and their causes and effects as nodes. Moreover, the logical relations between failure modes, and their causes and effects, are mapped as edges, for each edge may have different impact on other nodes, and we set weighted value to represent the difference.

### 3.2. The Construction of Weighted Network

According FMECA, we should first determine the indenture levels dependent on the functional relationship or composition characteristics of products. Generally, it can be divided into three layers, which are initial indenture level, other indenture level and lowest indenture level. Suppose that n and m are the numbers of failure modes, and their causes and effects and indenture levels, respectively, then the failure set and indenture level set are written as ${F\;=\;\{f}_{1}{,\;f}_{2}{,\;\ldots \;,\;f}_{\beta }\}$ and $D\;=\;\left\{d_{1}{,\;d}_{2}{,\;\ldots ,\;d}_{\alpha }\right\}$. For any two nodes, the coupling relation between $f_{i}$ and $f_{j}$ is denoted as $l_{\mathrm{ij}}$. So the coupling set is represented as ${L\;=\;\{l}_{\mathrm{ij}}|1\;\leq \;i\;\leq \;n,\;1\;\leq \;j\;\leq \;n\}$. If there exists node *i* and node *j* directly connected with edge $e_{\mathrm{ij}}$, then $l_{\mathrm{ij}}=\;1$, otherwise $l_{\mathrm{ij}}=\;0$. In addition, the set of weighted values is denoted as W, which indicates the force of a node on other nodes. In traditional FMECA, occurrence (*O*) and detection (*D*) have a certain overlap in the information. Generally speaking, it is easily to be detected for the failure mode if the probability of occurrence is large. Therefore, in order to eliminate the redundancy of information, according to the meaning of failure modes, and their causes and effects, combined with the influence relations between them, $\sqrt{O_{\mathrm{ij}}\cdot D_{\mathrm{ij}}}$ is represented as the weight of failure cause to failure mode in this paper, and $S_{ij}$ is used to weight the influence of that failure mode on failure effect. So we represent the FMECA model as the following.

**Figure 2:**
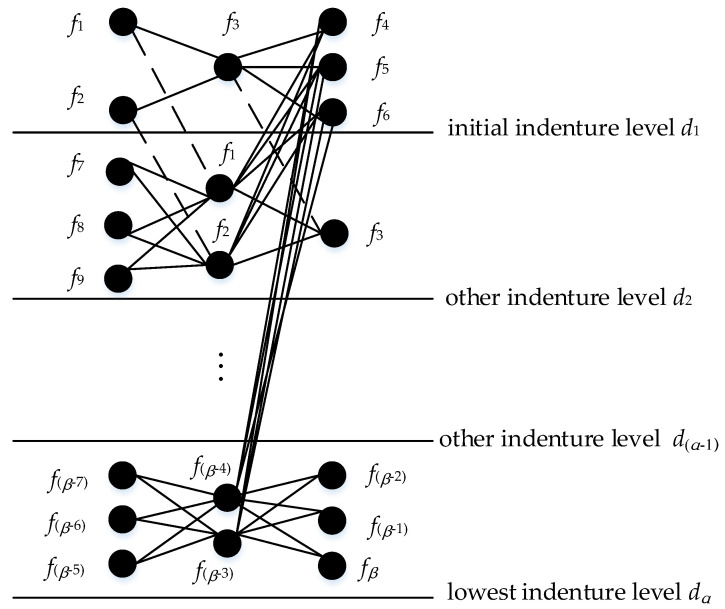
A case of structure diagram of the Failure Mode, Effects and Criticality Analysis (FMECA).

*G* = (*N*, *E*, *W*) (2)

where, $N\;=\;\left\{N_{1}{,\;N}_{2}{,\;\ldots \;,\;N}_{n}\right\}{,\;E\;=\;\{e}_{11}{,\;e}_{12}{,\;\ldots \;e}_{\mathrm{ij}}{,\;\ldots \}}_{n\times n}{,\;W\;=\;\{w}_{11}{,\;w}_{12}{,\;\ldots \;w}_{\mathrm{ij}}{,\;\ldots \}}_{n\times n}$(1 ≤ i ≤ n, 1 ≤ j ≤ n) denote the nodes set, edges set and weights set, respectively. Particularly, $w_{\mathrm{ij}}$ is represented by $\sqrt{O_{\mathrm{ij}}\cdot D_{\mathrm{ij}}}$ or $S_{ij}$. Based on the logical relationships at different levels of products in Figure 1, we propose a complex network structure diagram as shown in Figure 2. The steps to determine the entire structure diagram are as follows:

Step 1: According to the quality control needs of products, determine the initial indenture level $d_{1}$, find failure modes of level $d_{1}$ and determine their causes and effects.

Step 2: The failure causes of initial the indenture level $d_{1}$ is respectively corresponding to the failure modes of the other indenture level $d_{2}$, and the failure modes of $d_{1}$ are mapped as the failure effects of the other indenture level $d_{2}$, respectively; furthermore, determine failure causes of level $d_{2}$.

Step 3: In the same way, continue to search for failure modes and their causes and effects of the next level, until all of them are searched; denote this level as the lowest indenture level $d_{\alpha }$.

Step 4: Searching up from the lowest indenture level $d_{\alpha }$, layer by layer until all fault modes are no longer matched to the failure causes of the other level.

Step 5: Finally, all the failure modes and their causes and effects are respectively mapped to nodes, and the relationship existing between them is converted into an edge, and only one node is reserved for the recurring nodes. Accordingly, a structure diagram of FMECA is established as shown in Figure 2.

In Figure 2, {$f_{1}{,\;f}_{2}{,\ldots ,\;f}_{\beta }$} is represented to failure modes, and their causes and effects set. The black solid line is used to describe the direct coupling relation and the dash line indicates that failure modes, and their causes and effects, correspond to each other in different levels; each black solid line is given a weight $w_{\mathrm{ij}}$.

**Figure 3:**
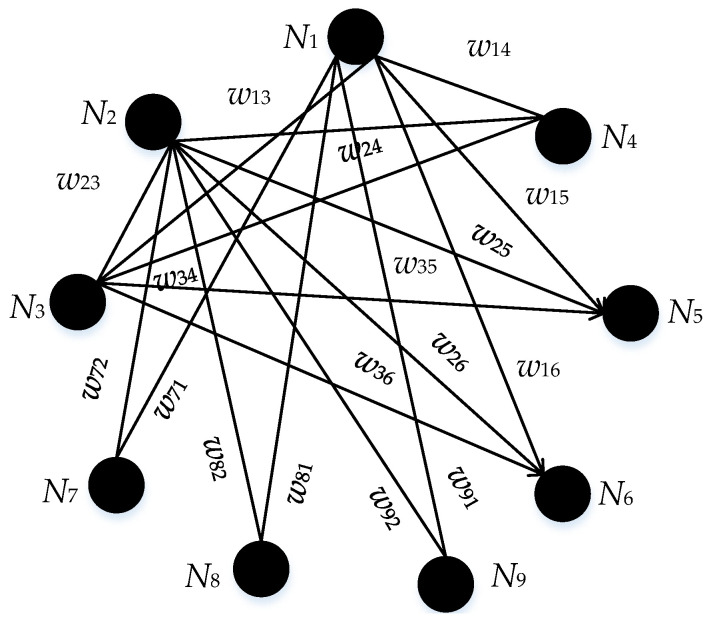
An example of a weighted network.

Assume that there are only the initial indenture level $d_{1}$ and the lowest indenture level $d_{2}$ in Figure 2. Then an example of a weighted network is set up in Figure 3.

As shown in Figure 3, $\left\{N_{1}{,\;N}_{2}{,\ldots ,\;N}_{9}\right\}$ and {$f_{1}{,\;f}_{2}{,\ldots ,\;f}_{9}$} is one-to-one correspondence. The values of weights, denoted as $w_{\mathrm{ij}}$, are listed in Table 1.

### 3.3. Information Entropy and Algorithm

Information entropy is widely used in information science and statistical physics to describe the order of information distribution [39]. If X is a set of possible events $x_{1}$, $x_{2}$, …,${\;x}_{n}$, and $p_{i}$ is the probability of $x_{i}$, the entropy of X can be calculated as:

(3) $$E(X)\;=\;-\overset{n}{\underset{i\;=\;1}{\sum }}p_{i}\log f_{0}(p_{i})$$

where, $0\;\leq \;p_{i\;}\leq \;1$, $\sum_{\;i=\;1}^{n}p_{i}$ = 1. On the one hand, when the values of *n* are equal, if the probabilities $p_{i}$ have uniform distribution, then the higher the entropy value will be. Additionally, as the value of *n* increases, so does the entropy value. Therefore, employing entropy can be useful for the detection of nodes with high-degree, more uniform neighbors.

Dehmer suggested introducing a tuple ($\lambda_{1}{,\;\lambda }_{2}{,\;\ldots ,\;\lambda }_{n}$) of non-negatives in order to form a probability distribution ${p\;=\;(p}_{1}{,\;p}_{2}{,\ldots ,\;p}_{n})$ which is described as follows [40].

(4) $$p_{i}\;=\;\frac{\lambda_{i}}{\sum_{j\;=\;1}^{n}\lambda_{j}}i\;=\;1,\;2,\ldots ,\;n$$

In Equation (4), $\lambda_{i}$ represents the *i*th non-negative integer. Therefore, the Equation (4) can be written as

(5) $$E\left(X\right)\;=\;\log\left(\sum_{i=1}^{n}\lambda_{i}\right)-\sum_{i\;=\;1}^{n}\frac{\lambda_{i}}{\sum_{j\;=\;1}^{n}\lambda_{j}}{\log\lambda }_{i}$$

**Figure 4:**
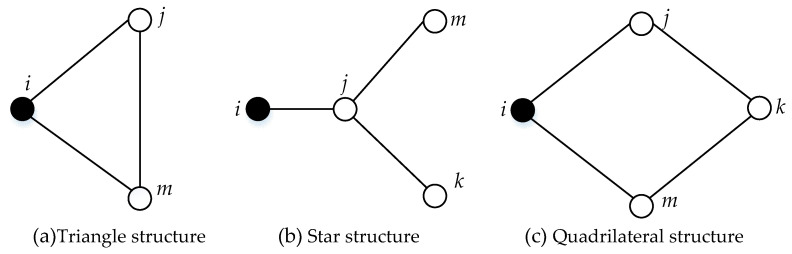
Argument structure in neighborhoods network, which is included as: (**a**) node *j* and *m* are first-order neighbors of node *i*; (**b**) node *j* is first-order neighbor of node *i*, and node *m* and *k* are second-order neighbors of node *i*; (**c**) node *j* and *m* are first-order neighbors of node *i*, and node *k* is the second-order neighbors of node *i*.

In addition, we find that a neighborhood network of FMECA usually concludes three argument structures, which are showed in Figure 4.

Particularly, in a complex network for FMECA, there is a direct connection between fault cause and fault mode, and fault mode link to fault effect as well. Moreover, a correspondence between different indenture level makes a number of vertices directly linked together; the second order or above neighbor node in the network is quite rare. Based on the above analysis, although research has shown that the entropy centrality should have higher precision if considering the influence of the second order or above neighbor node, this paper calculates entropy centrality and only considers the first neighbor node.

In this paper, we take advantages of both topological structure and information entropy, where the local power of a given vertex includes not only structural entropy but also interaction frequency entropy [41]. The structural entropy evaluates the influence or strength of a given node based on the topographic properties of the sub-graph. Similarly, the interaction frequency entropy, which takes advantage of information contained in the weights of edges that rest between nodes and nodes, depicts the propagation effectiveness of a given node.

Additionally, the degree of node *i* is represented as ${\mathrm{DC}}_{i}$, and expresses the influence on the neighbor nodes,

(6) $${\mathrm{DC}}_{i}\;=\;\sum_{j\in n}e_{\mathrm{ij}},\;j\;\neq \;i$$

where, $e_{\mathrm{ij}}$ which denotes the node *i* has a direct link to the neighbor node *j.* In addition, we denote *M* is the number of first order neighbors of node *i.* Thus, we obtain the tuple ($\lambda_{1}{,\;\lambda }_{2}{,\ldots \;,\;\lambda }_{M\;+\;1}$), and define it as follows.

(7) $$\lambda_{i}{\;=\;\mathrm{DC}}_{i}$$

Based on the above definition, we obtain that the structural entropy centrality of node *i* is denoted by ${\mathrm{EC}}_{i}^{s}$:

(8) $${\mathrm{EC}}_{i}^{s}\;=\;\log\left(\sum_{i\;=\;1}^{M\;+1}{\mathrm{DC}}_{i}\right)-\sum_{i\;=\;1}^{M\;+1}\frac{{\mathrm{DC}}_{i}}{\sum_{i\;=\;1}^{M\;+\;1}{\mathrm{DC}}_{i}}{\log\mathrm{DC}}_{i}$$

In addition, the weight of edges plays a role in estimating the interaction frequency, we denote ${\mathrm{EC}}_{i}^{f}$ as the interaction frequency entropy of node *i*, and it is defined as:

(9) $${\mathrm{EC}}_{i}^{f}\;=\;-\sum_{j\;=\;1}^{M}\log\frac{W_{\mathrm{ij}}}{\sum_{k\;=\;1}^{M}W_{\mathrm{ik}}}\log\frac{W_{\mathrm{ij}}}{\sum_{k\;=\;1}^{M}W_{\mathrm{ik}}}$$

where $W_{\mathrm{ij}}$ denotes the weight of the edge and *M* indicates the number of first order neighbors of node *i*. Combining ${\mathrm{EC}}_{i}^{s}$ with ${\mathrm{EC}}_{i}^{f}$, denoted as ${\mathrm{EC}}_{i}$, which equals the summation of the structural entropy and frequency entropy, multiplied by two parameters, respectively, so the ${\mathrm{EC}}_{i}$ is computed through the following equation:

(10) $${\mathrm{EC}}_{i}{\;=\;\xi }_{1}{\mathrm{EC}}_{i\;}^{s}{\;+\;\xi }_{2}{\mathrm{EC}}_{i}^{f}$$

where $\xi_{1}$ and $\xi_{2}$ stand for the weight coefficients, respectively, and $\xi_{1}+\xi_{2\;}=\;1.$

### 3.4. A Summary of Method

We present a novel method for supporting FMECA that introduces complex theory into FMECA, and failure modes, and their causes and effects are denoted as nodes, while the edges represent nodes connected to the other nodes. In addition, the edge between failure cause and failure mode is weighted as $\sqrt{\mathrm{OD}}$, and the link for that failure mode with failure effect is denoted as S. By mining topological structure in complex networks, and making use of information contained in the weighted links, we finally get an improved RPN indicator, which is ${\mathrm{EC}}_{i}$, and it is named as ECRPN, and means a way to evaluate RPN by entropy centrality. Moreover, the weight of each edge will change as long as any indicator of the *O*, *S*, *D* changes. Besides, with the improvement of social management and technology, when people discover there is no longer any influence relation on certain failure modes, and their causes and effects, or some failure modes, and their causes and effects no longer affect the product quality, some corresponding vertices or edges will also disappear. Therefore, the weighted graph which we established is dynamic. Further, values for $\xi_{1}$ and $\xi_{2}$ are purposely set as 0.4 and 0.6, respectively. Peng et al. [42] have demonstrated that entropy-based centrality outperformances the classic degree-based centralities and path-based centralities under the conditions of this particular set of parameters. Thus, the Equation (10) is denoted as:

(11) $${\mathrm{EC}}_{i}\;=\;-0.4\sum_{j\;=\;1}^{M+\;1}\frac{{\mathrm{DC}}_{i}}{\sum_{i\;=\;1}^{M\;+\;1}{\mathrm{DC}}_{i}}\log\frac{{\mathrm{DC}}_{i}}{\sum_{i\;=\;1}^{M\;+\;1}{\mathrm{DC}}_{i}}-0.6\sum_{j\;=\;1}^{M}\frac{W_{\mathrm{ij}}}{\sum_{k\;=\;1}^{M}W_{\mathrm{ik}}}\log\frac{W_{\mathrm{ij}}}{\sum_{k\;=\;1}^{M}W_{\mathrm{ik}}}\;\leq \;0.4\log\left(M\;+\;1\right)\;+\;0.6\log M$$

where, *M* is the number of node *i*’s neighbors. Furthermore, a specific safe threshold is set to screen for nodes with high risk, and the safe threshold is 70% of the maximum value of the entropy centrality of each node. It is represented as the Equation (12).

(12) $${\mathrm{EC}}_{i}^{\mathrm{safe}}\;=\;0.28\;\log\left(M\;+\;1\right)\;+\;0.42\;\log\;M$$

**Figure 5:**
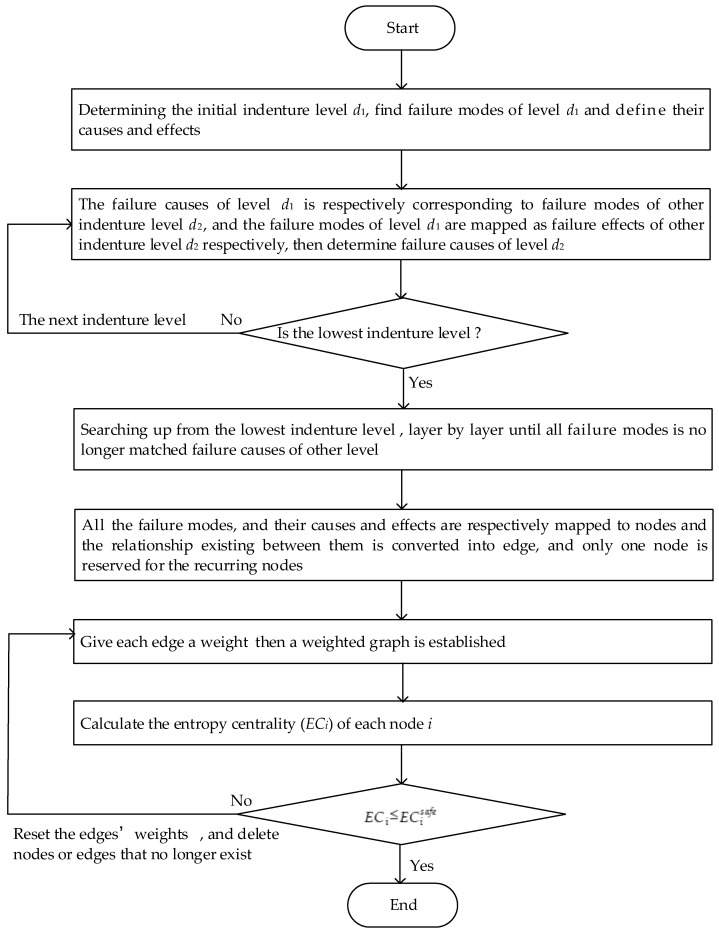
The flow of the whole proposed method.

According to the above description, the flow of the whole method we proposed is shown in Figure 5.

## 4. Case Study

**Figure 6:**
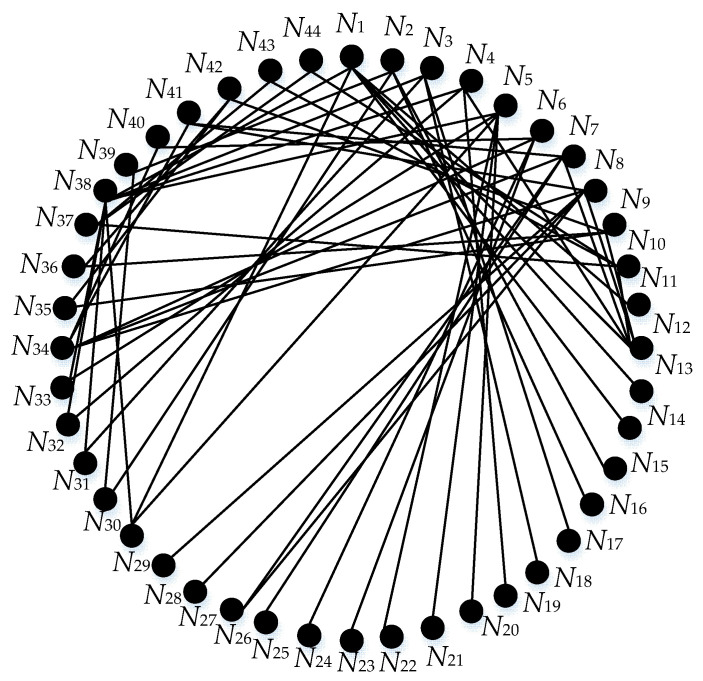
The weighted graph of HVAC system.

**Figure A1:**
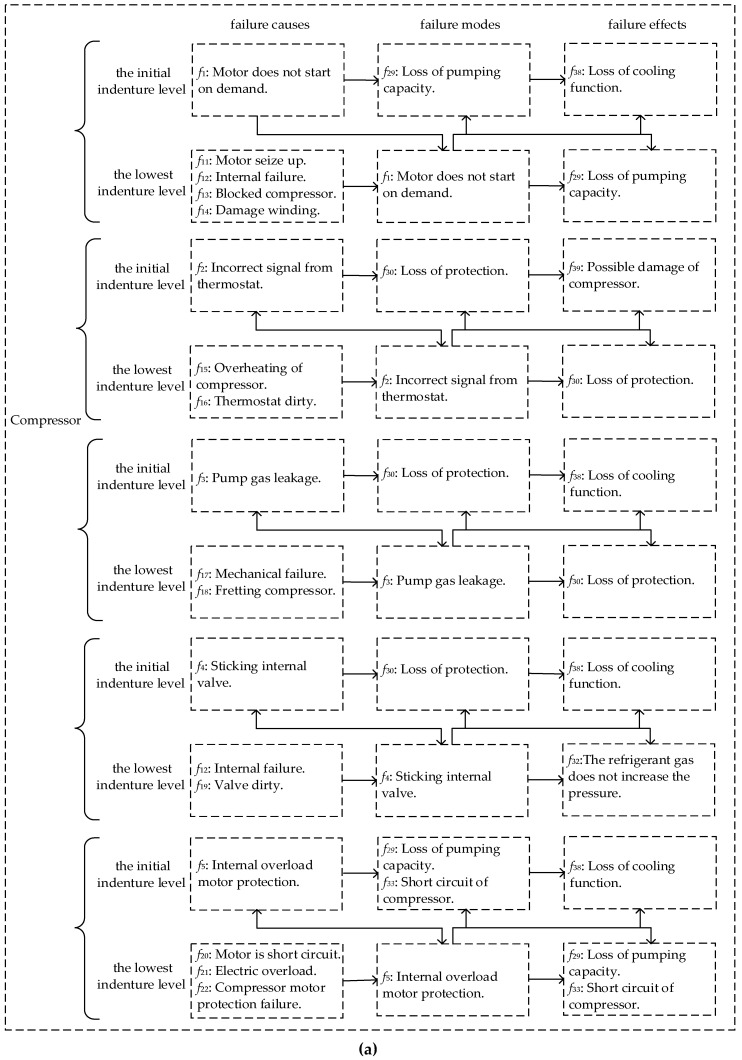

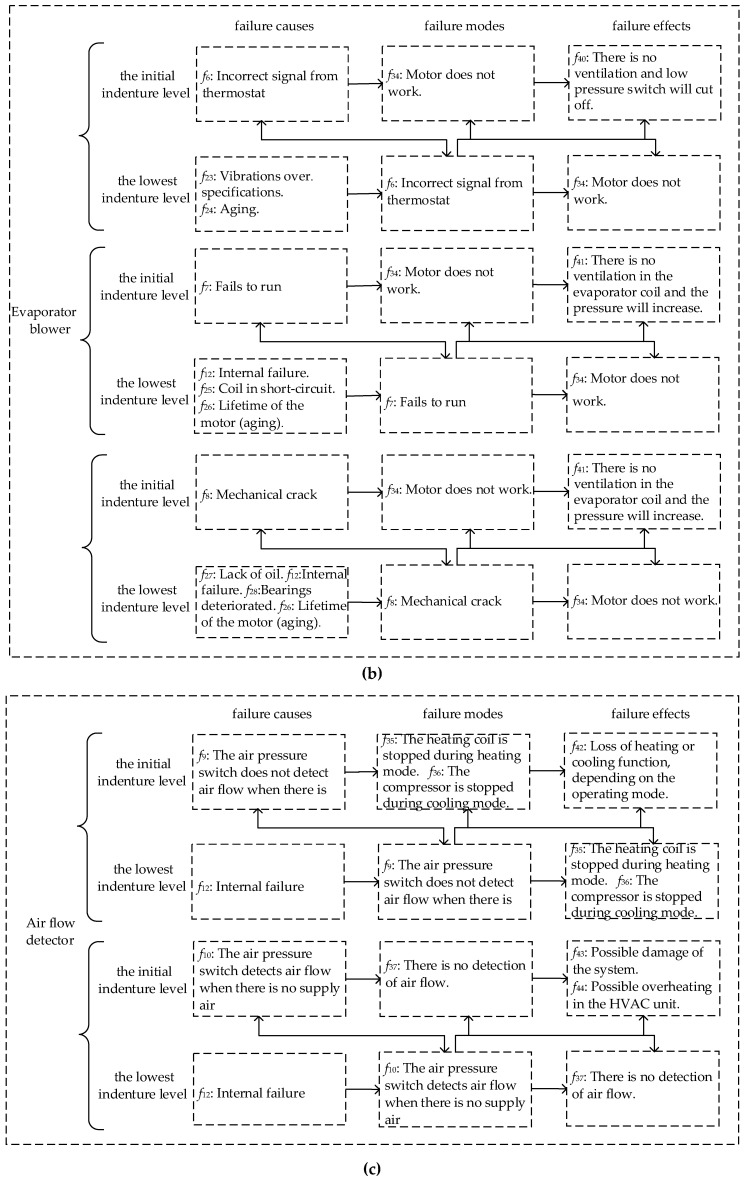
Failure modes and effects analysis for the HVAC system, which include three most critical components: (**a**) compressor; (**b**) evaporator blower; (**c**) air flow detector.

A real-world case about the Heating, Ventilation and Air Conditioning (HVAC) system was analyzed by FMECA, which came from Ciani et al.’s study [7]. Ciani et al. focused on some of the most critical components that make up the HVAC: compressor, evaporator blower and air flow detector. According to the actual situation, the component is regarded as the lowest indenture level. In addition, based on the difference of product hardware, the system is regarded as the initial indenture level. Hence, we can divide the system into two indenture levels, where the HVAC system is the initial indenture level $d_{1}$, and the components are the lowest indenture level $d_{2}$. All the failure modes, and their causes and effects, are shown in Appendix A Figure A1, based on the method we proposed, and the weighted graph is shown in Figure 6.

Moreover, the weight between nodes and the nodes’ degrees are included in Appendix A Table A1. Further, we take the node 1 as an example to describe the calculating process of the proposed algorithm. On the basis of Equation (8), if 10 is the base of the logarithmic function, then the structural entropy of node 1 is computed as follows:

(13) $${\mathrm{EC}}_{1}^{s}\;=\;\log\left(\sum_{\;i\;=1}^{7}{\mathrm{DC}}_{i}\right)-\sum_{\;i\;=1}^{7}\frac{{\mathrm{DC}}_{i}}{\sum_{i\;=\;1}^{7}{\mathrm{DC}}_{i}}{\log\mathrm{DC}}_{i\;}\;=\;0.726$$

Furthermore, following Equation (9), the interaction frequency entropy is expressed as follows:

(14) $${\mathrm{EC}}_{1}^{f}\;=\;-\sum_{j\;=\;1}^{6}\log\frac{W_{\mathrm{ij}}}{\sum_{k\;=\;1}^{6}W_{\mathrm{ik}}}\log\frac{W_{\mathrm{ij}}}{\sum_{k\;=\;1}^{6}W_{\mathrm{ik}}}\;=\;0.7759$$

And according to the Equation (10), we finally obtain the improved RPN indicator, represented by ${\mathrm{EC}}_{1}$, which is stated as follows:

(15) $${\mathrm{EC}}_{1}\;=\;0{.4\mathrm{EC}}_{1}^{s}\;+\;0{.6\mathrm{EC}}_{1}^{f}\;=\;0.7562$$

As discussed above, based on the entropy centrality approach, the power of each node and the corresponding results are recorded in Table 2.

**Figure 7:**
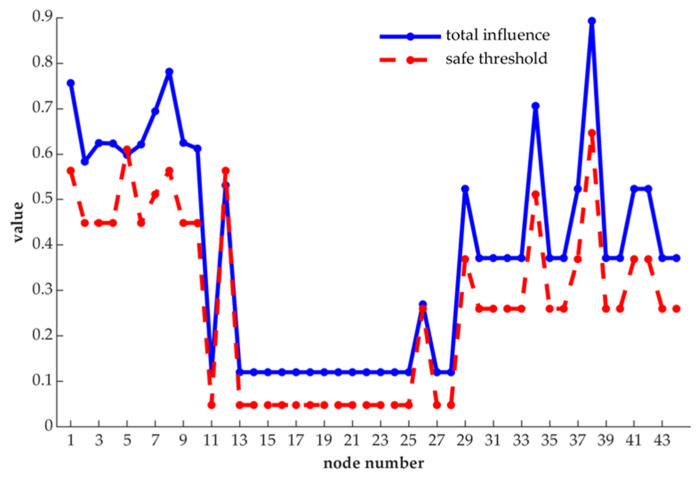
Comparison results of each node’s total influence and safe threshold.

Further, the results obtained from the analysis of our proposed method are compared with each node’s safe threshold, and we present the result in Figure 7.

In addition, we compare the results with traditional RPN method and alternative RPNs method, the difference is shown in Table 3.

As shown in Figure 7, in the whole network, node 1, node 8, node 34 and node 38 are obviously more important than other nodes, for their changes can have a greater impact on the nature of the network, and they can affect more nodes, too. Besides, excepting node 5 and node 12, the remaining nodes have entropy centrality values that exceed their safe threshold. Therefore, these nodes whose entropy centrality values are more than the safe thresholds should be taken as control measures.

Moreover, in Table 3, it is shown that node 7 has the highest risk priority number based on the traditional RPN method, but it is not among the four most important nodes, it is ranked fifth place. Although node 1 and node 8 are ranked third and fourth floor with the classical RPN method, but their neighbor nodes are all 6, and the neighbor nodes of node 7 are 5, and lastly node 1 and node 8 are more influential than node 7 in the weighted network. On the one hand, nodes 1 and 8 have more neighbor nodes than node 7, which indicates that nodes 1 and 8 have more functional coupling relationships with other nodes. Through functional coupling, components compressor and evaporator blower complete certain specified actions, respectively. On the other hand, the parameters *O* and *D* overlap in information, *O* and *D* of node 1 are 8 and 7, respectively, *O* and *D* of node 7 are 9 and 6, and *O* and *D* of node 8 are 8 and 5, respectively. If traditional FMECA is used, the results for nodes 1, 7 and 8 are 56, 54 and 40, respectively. The parameters S of nodes 1, 7 and 8 are 6, 8 and 8, respectively. By combining the results of filtering the information of parameters *O* and *D*, and the size of parameter *S*, obviously, the results of ECRPN are more scientific. Moreover, for HVAC systems, more than half of the failures are caused by electrical reasons, while nearly 20% of failures are mechanical faults, and a small part is caused by pipelines and switches, and about 85% of electrical failures are caused by motors which do not start on demand, and considering the effect caused by the failure, and the difficulty of detecting failure, the actual situation shows that the ranking of ECRPN is closer to the actual situation.

## 5. Conclusions and Discussion

This paper proposed a novel approach of introducing complex network theory into FMECA. The approach starts from the need to transform traditional FMECA into a weighted complex network. So initially we determined indenture levels that depended on the functional relationship or composition characteristics of products. Then, we use traditional FMECA to identify all failure modes, the causes and effects of each mode of the system. Next, we defined failure modes, and their causes and effects as nodes, and the logical relation of nodes are denoted as edges, where the edge between failure cause and failure mode is weighted as $\sqrt{\mathrm{OD}}$, and the link of that failure mode with failure effect is denoted as S. Then a weighted graph is established. Furthermore, the entropy centrality approach is applied to rank influential nodes. Finally, a real-world case is presented to illustrate and verify the proposed method. The results show that considering the logical relationship between the failure modes, and their causes and effects, it does have an impact on the failure mode ranking.

In this study, we use the entropy centrality method to estimate the influential nodes of the weighted graph we proposed, obviously, perfect algorithms do not exist without any limitations or assumptions. Thus, as for future work, we expect to carry out further work on improving entropy-based centrality. Moreover, the work presented in this article does not consider the difference among the local influence on the layer, the high-level impact on the upper adjacent layer, and the final impact on the initial indenture level. The magnitude of these three effects is represented by the weight *S*, which may not match the real-world situation. In addition, this method is based on complex networks to mine the relation between failure information. It may not be applicable to systems with insufficient failure information. Thus, our proposed method could be enhanced in the future, making it more applicable.

## Figures and Tables

**Table 1:** The weights of one node to another.

Between Two Nodes	Weight	Between Two Nodes	Weight
1–3	$\sqrt{O_{13}D_{13}}$	3–5	$S_{35}$
1–4	$S_{14}$	3–6	$S_{36}$
1–5	$S_{15}$	7–1	$\sqrt{O_{71}D_{71}}$
1–6	$S_{16}$	7–2	$\sqrt{O_{72}D_{72}}$
2–3	$\sqrt{O_{23}D_{23}}$	8–1	$\sqrt{O_{81}D_{81}}$
2–4	$S_{24}$	8–2	$\sqrt{O_{82}D_{82}}$
2–5	$S_{25}$	9–1	$\sqrt{O_{91}D_{91}}$
2–6	$S_{26}$	9–2	$\sqrt{O_{92}D_{92}}$
3–4	$S_{34}$		

**Table 2:** The results of entropy centrality of each node and the corresponding ranking results.

Node	${\mathrm{EC}}_{i}^{s}$	${\mathrm{EC}}_{i}^{f}$	Total Influence	No.	Node	${\mathrm{EC}}_{i}^{s}$	${\mathrm{EC}}_{i}^{f}$	Total Influence	No.
1	0.7266	0.7759	0.7562	3	23	0.1204	0	0.1204	17
2	0.6582	0.5337	0.5835	12	24	0.1204	0	0.1204	17
3	0.6582	0.6017	0.6243	7	25	0.1204	0	0.1204	17
4	0.6582	0.6003	0.6234	8	26	0.4515	0.1485	0.2697	16
5	0.7476	0.4989	0.5984	11	27	0.1204	0	0.1204	17
6	0.6582	0.5966	0.6213	9	28	0.1204	0	0.1204	17
7	0.6876	0.6986	0.6942	5	29	0.5933	0.4771	0.5236	14
8	0.7266	0.8175	0.7812	2	30	0.4771	0.3010	0.3714	15
9	0.6589	0.6020	0.6248	6	31	0.4771	0.3010	0.3714	15
10	0.6589	0.5811	0.6122	10	32	0.4711	0.3010	0.3714	15
11	0.1204	0	0.1204	17	33	0.4711	0.3010	0.3714	15
12	0.6901	0.7702	0.5315	13	34	0.7611	0.6990	0.7060	4
13	0.1204	0	0.1204	17	35	0.4711	0.3010	0.3714	15
14	0.1204	0	0.1204	17	36	0.4711	0.3010	0.3714	15
15	0.1204	0	0.1204	17	37	0.5933	0.4771	0.5236	14
16	0.1204	0	0.1204	17	38	0.8785	0.9024	0.8929	1
17	0.1204	0	0.1204	17	39	0.4771	0.3010	0.3714	15
18	0.1204	0	0.1204	17	40	0.4771	0.3010	0.3714	15
19	0.1204	0	0.1204	17	41	0.5933	0.4771	0.5236	14
20	0.1204	0	0.1204	17	42	0.5933	0.4771	0.5236	14
21	0.1204	0	0.1204	17	43	0.4771	0.3010	0.3714	15
22	0.1204	0	0.1204	17	44	0.4771	0.3010	0.3714	15

**Table 3:** Failure mode of the Heating, Ventilation and Air Conditioning (HVAC) system ranked using traditional and alternative risk priority numbers (RPNs).

Failure Mode	O	S	D	RPN		IRPN		ERPN		URPN		LRPN		ECRPN	
				Value	Rank	Value	Rank	Value	Rank	Value	Rank	Value	Rank	Value	Rank
*f*_1_	8	6	7	336	3	21	2	9477	4	22,592	5	2527	3	0.7562	2
*f*_2_	3	5	4	60	10	12	10	351	10	1780	9	1778	10	0.5835	10
*f*_3_	6	6	7	252	6	19	6	3645	7	16,944	6	2401	6	0.6243	5
*f*_4_	5	6	5	150	7	16	8	1215	8	10,085	7	2176	7	0.6234	6
*f*_5_	4	6	5	120	9	15	9	1053	9	8068	8	2079	9	0.5984	9
*f*_6_	8	8	4	256	5	20	5	13,203	3	95,390	3	2408	5	0.6213	7
*f*_7_	9	8	6	432	1	23	1	26,973	1	160,971	1	2636	1	0.6942	3
*f*_8_	8	8	5	320	4	21	2	13,365	2	119,238	2	2505	4	0.7812	1
*f*_9_	7	7	7	343	2	21	2	6561	5	53,735	4	2535	2	0.6248	4
*f*_1z_	7	3	7	147	8	7	7	4401	6	984	10	2167	8	0.6122	8

## Appendix A

**Table A1:** The weight between nodes and the degrees of nodes.

Between Two Nodes	Weight	${\mathrm{DC}}_{i}$	Between Two Nodes	Weight	${\mathrm{DC}}_{i}$
1–29	$\sqrt{56}$		17–3	$\sqrt{42}$	1
1–38	6		18–3	$\sqrt{42}$	1
11–1	$\sqrt{56}$		19–4	5	1
12–1	$\sqrt{56}$	6	20–5	$\sqrt{20}$	1
13–1	$\sqrt{56}$		21–5	$\sqrt{20}$	1
14–1	$\sqrt{56}$		22–5	$\sqrt{20}$	1
2–30	$\sqrt{12}$		23–6	$\sqrt{32}$	1
2–39	5	4	24–6	$\sqrt{32}$	1
15–2	$\sqrt{12}$		25–7	$\sqrt{54}$	1
16–2	$\sqrt{12}$		26–7	$\sqrt{54}$	2
3–31	$\sqrt{42}$		26–8	$\sqrt{40}$	
3–38	6	4	27–8	$\sqrt{40}$	1
17–3	$\sqrt{42}$		28–8	$\sqrt{40}$	1
18–3	$\sqrt{42}$		29–38	6	
4–32	$\sqrt{25}$		1–29	$\sqrt{56}$	3
4–38	6		5–29	$\sqrt{20}$	
12–4	$\sqrt{25}$	4	30–39	5	2
19–4	$\sqrt{25}$		2–30	$\sqrt{12}$	
5–29	$\sqrt{20}$		31–38	6	2
5–33	$\sqrt{20}$		3–31	$\sqrt{42}$	
5–38	6	6	32–38	6	2
20–5	$\sqrt{20}$		4–32	5	
21–5	$\sqrt{20}$		33–38	6	2
22–5	$\sqrt{20}$		5–33	$\sqrt{20}$	
6–34	$\sqrt{32}$		34–40	8	
6–40	8	4	34–41	8	
23–6	$\sqrt{32}$		6–34	$\sqrt{32}$	5
24–6	$\sqrt{32}$		7–34	$\sqrt{54}$	
7–34	$\sqrt{54}$		8–34	$\sqrt{40}$	
7–41	8		35–42	7	2
12–7	$\sqrt{54}$	5	9–35	7	
25–7	$\sqrt{54}$		36–42	7	2
26–7	$\sqrt{54}$		9–36	7	
8–34	$\sqrt{40}$		37–43	3	
8–41	8		37–44	3	3
12–8	$\sqrt{40}$	6	10–37	7	
26–8	$\sqrt{40}$		1–38	$\sqrt{56}$	
27–8	$\sqrt{40}$		3–38	$\sqrt{42}$	
28–8	$\sqrt{40}$		4–38	5	
9–35	7		5–38	$\sqrt{20}$	8
9–36	7	4	29–38	6	
9–42	7		31–38	6	
12–9	7		32–38	6	
10–37	7		33–38	6	
10–43	3	4	2–39	$\sqrt{12}$	2
10–44	3		30–39	5	
12–10	7		6–40	8	2
11–1	$\sqrt{56}$	1	34–40	8	
12–1	$\sqrt{56}$		7–41	8	
12–4	5		8–41	8	3
12–7	$\sqrt{54}$	6	34–41	8	
12–8	$\sqrt{40}$		9–42	7	
12–9	7		35–42	7	3
12–10	7		36–42	7	
13–1	$\sqrt{56}$	1	10–43	3	2
14–1	$\sqrt{56}$	1	37–43	3	
15–2	$\sqrt{12}$	1	10–44	3	2
16–2	$\sqrt{12}$	1	37–44	3	
